# Blood Microbiome: A New Marker of Gut Microbial Population in Dogs?

**DOI:** 10.3390/vetsci7040198

**Published:** 2020-12-04

**Authors:** Elisa Scarsella, Misa Sandri, Simeone Dal Monego, Danilo Licastro, Bruno Stefanon

**Affiliations:** 1 Department of Agriculture, Food, Environmental and Animal Science, University of Udine, 33100 Udine, Italy; scarsella.elisa@spes.uniud.it (E.S.); misa.sandri@uniud.it (M.S.); 2 ARGO Open Lab Platform for Genome Sequencing, AREA Science Park, Padriciano, 34149 Trieste, Italy; simeone.dalmonego@areasciencepark.it (S.D.M.); danilo.licastro@areasciencepark.it (D.L.)

**Keywords:** microbiota, blood, gut, dog, healthy, nutrition

## Abstract

The characterization of the microbial population in different compartments of the organism, such as the gastrointestinal tract, is now possible thanks to the use of high-throughput DNA sequencing technique. Several studies in the companion animals field have already investigated the fecal microbiome in healthy or sick subjects; however, the methodologies used in the different laboratories and the limited number of animals recruited in each experiment do not allow a straight comparison among published results. Previously, our research focused on the characterization of the microbial taxa variability in 340 fecal samples from 132 healthy dogs, collected serially from several in-house experiments. The results supported the responsiveness of microbiota to dietary and sex factors and allowed us to cluster dogs with high accuracy. For the present study, intestinal and blood microbiota of healthy dogs from different breeds, genders, ages and food habits were collected, with three principal aims: firstly, to confirm the results of our previous study regarding the fecal microbiome affected by the different type of diet; secondly, to investigate the existence of a blood microbial population, even in heathy subjects; and thirdly, to seek for a possible connection between the fecal and the blood microbiota. Limited researches have been published on blood microbiota in humans, and this is the first evidence of the presence of a bacterial population in the blood of dogs. Moreover, gut and blood microbiota can discriminate the animals by factors such as diet, suggesting some relationship between them. These preliminary results make us believe in the use of the blood microbiome for diagnostic purposes, such as researching and preventing gut inflammatory diseases.

## 1. Introduction

The numerous studies that investigated the composition and the variation of gut microbiome in relation to healthy conditions and environmental factors for companion animals and livestock have attracted the scientific community and are growing exponentially [1,2]. The microbial population that inhabits the gastrointestinal tract of both humans and animals has been considered responsible of very important basic functions, from the regulation of metabolic activities to protection against pathogens and modulation of immune system and then physiologic functions [3]. The advent of innovative technologies allows for a more frequent utilization of molecular methodologies to identify non-culturable bacteria within the canine gut, highlighting the high individual variability of microbial population, especially in dietary intervention studies [4,5]. Despite these evidences, it appears clear that some key bacterial species are consistently present in fecal samples of healthy subjects, suggesting the presence of a core fecal bacterial community [6].

In a recent study on healthy dogs, the paramount role of diet and sex on the gut microbiome has been reported [7], confirming the idea that the microbes inhabiting the gut can be considered as an individual fingerprint [8]. Human microbiome can be split in enterotypes, meaning that individuals can be clustered on the basis of the abundance of microbial taxa of the gut [9]. The categorization in enterotypes has not yet been applied to dogs, also because the contradictory results that can be found in the literature make the identification of a gut microbiome core in dogs difficult. Interestingly, Scarsella et al. (2020) [7] showed that dogs fed with a homemade diet (H) cluster together with dogs fed with a raw-based diet supplemented with a complementary food (B.A.S.E., www.nutrigenefood.com., Italy), even though the composition of these two diets was different. On the opposite, dogs fed with a commercial extruded complete diet (K) and a commercial moist complete diet (W) formed two distinctly clusters. It is likely that the presence of raw meat in the former diets and the physical form of the diets had a similar impact on shaping gut microbiome. Similar results have been obtained analyzing the gut microbiome in relation to the sex factor.

The prevalence of specific taxa can be effective to identify dysbiosis events. The concept of “dysbiosis” refers to a change in the composition of symbiotic or commensal microbial communities [10]. Considerable research has been dedicated to address the relationship between the gut microbiome and the health status of the subjects, both human and animals, but there is still a limited number of studies, only in the human field, that explored dysbiosis related to the blood microbiome and its potential role in pathogenesis. Blood has been traditionally considered to be a sterile environment, but some evidences in various domesticated mammals and birds [11,12] and in humans [13,14,15] suggest that it is populated by microbes. The origin of these bacteria is mainly attributed to the translocation from the gastro-intestinal tract [16], but it has been suggested that also a part of the microbial population of the oral cavity and the skin can diffuse into the blood [17]. The hypothesis is that many bacteria found in healthy human blood may be in a dormant state [18], or they are present in their L-forms [19].

The present study has three principal aims: firstly, to confirm the results of our previous study regarding the role of diet on fecal microbiome; secondly, to investigate the presence of a microbial population in the bloodstream in heathy subjects’ and thirdly, to seek for a possible connection between the fecal and the blood microbiota.

## 2. Materials and Methods

### 2.1. Animals and Housing

Thirty-six healthy dogs were enrolled from three veterinary clinics. Both females (20 dogs, 10 of which were spayed) and males (16 intact dogs) were present in this study, and the dogs were of different breeds (small- to medium-size) and in the adult phase (more than two years old, less than 10). Dogs were divided on the basis of the type of diet consumed regularly since at least three months before the visit. The first group (10 dogs) was fed with commercial extruded complete dry pet foods (KIBBLE), the second group (16 dogs) with a raw meat diet (BARF) and the third group (10 dogs) with a homemade diet (HOME). The main ingredients of kibbles were chicken meat and fat, rice, and beet pulp, with an average crude protein content of 26.5% and fat content of 15.5% on a dry-matter basis. The BARF diet was made of a mix of meats (about 60% of beef or turkey or chicken, as fed), offal (about 20% as fed), bones (about 10% as fed), vegetables and oils (about 10% as fed). The HOME diet was formulated by a nutritionist, with an average of 45% raw beef meat, 40% boiled rice, 10% vegetables as fed and 5% mineral vitamins supplement. The dogs were presented to the clinics for normal routine visit, and the owners were asked to take the leftover feces and blood samples collected for the screening. Two clinics were situated in the city and were more generalist, and one was specialized and situated in the countryside. For each dog, one sample of blood and one sample of feces were collected the same day, to minimize the variability between these two types of biological matrices. An informed consent was obtained by the owners, and the dogs were housed in their usual home and condition. All protocols and procedures for the animals complied with the Italian legislation on animal care and were approved by the ethical committee of the University of Udine (OPBA, Prot. N. 7/2019, issued on 28 June 2019). Fecal material was transferred, using sterile gloves, into a sterile plastic bag and immediately stored at −20 °C until the analysis. Whole blood was collected in a K_3_-EDTA tube with venipuncture from the radial vein after shaving the coat and careful disinfected with chlorhexidine alcohol solution. The samples were immediately stored at −20 °C until the analysis.

### 2.2. Fecal and Blood DNA Extraction, Sequencing, and Taxonomic Annotation

Microbial DNA was extracted by following the instructions of two commercial kit, based on the starting material. DNA from fecal samples was extracted from 150 mg of starting material, using a Fecal DNA MiniPrep kit with a bead beating step (Zymo Research; Irvine, CA, USA), whilst DNA from blood samples was extracted from 200 μL of starting material, using a Exgene™ Clinic SV kit (GenAll Biotechnology, Seoul, Korea). A ZymoBIOMICS™ Microbial Community Standard (Zymo Research, Irvine, CA, USA) was used to assess the efficiency of the entire pipeline, from DNA extraction method to taxonomic annotation. The mock community contains eight bacterial species: *Pseudomonas aeruginosa* (4.2%), *Escherichia coli* (10.1%), *Salmonella enterica* (10.4%), *Lactobacillus fermentum* (18.4%), *Enterococcus faecalis* (9.9%), *Staphylococcus aureus* (15.5%), *Listeria monocytogenes* (14.1%) and *Bacillus subtilis* (17.4%); expected composition of the mock community was given by the manufacturer. DNA concentration was measured with a QubitTM 3 Fluorometer (Thermo Scientific; Waltham, MA, USA), and then the 16S rRNA of V3 and V4 regions were amplified for library preparation, adding also the indexes for sequencing, using a Nextera DNA Library Prep kit (Illumina; San Diego, CA, USA), following the manufacturer’s instruction and primers [20]. The resulting amplicons were sequenced with a NovaSeq 6000 (Illumina; San Diego, CA, USA) in 2 × 300 paired-end mode, following the standard procedures. The Quantitative Insights into Microbial Ecology (QIIME 2) [21] was used to process the raw sequences, which were uploaded to NCBI Sequence Read Archive (Bioproject ID PRJNA668368). After demultiplexing, sequenced reads that passed the quality check (Phred score ≥ 30) were annotated for 16S rRNA against the most recent Greengenes database (version gg.13_8.otus.tar.gz), with 99%identifying with reference sequences. Chimeras were also detected and then filtered from the reads, and the remaining sequences were clustered into exact sequence variants by using an open-reference approach in QIIME 2. This procedure is the preferred strategy for exact sequence variants picking in QIIME2, which includes taxonomy assignment, sequence alignment and tree-building steps.

### 2.3. Quantitative Real-Time PCR (qPCR)

Quantifications of total bacteria were evaluated by qPCR, using the oligonucleotides tested by AlShawaqfeh et al. (2017) [22]. All samples were run in triplicate. The DNA extracted from the ZymoBIOMICS™ Microbial Community Standard (Zymo Research, Irvine, CA, USA) was used as a positive control and for the quantification of the total 16S copies DNA/g bacteria.

SYBR-based qPCR assays were performed by following the run protocol reported by AlShawaqfeh et al. (2017) [22], with some modifications. Briefly, SYBR-based reaction mixtures (total 12.5 μL) contained 6.25 μL of Platinum™ SYBR™ Green qPCR SuperMix-UDG (Invitrogen, Carlsbad, CA, USA), 3.25 μL of water, 0.25 μL of each primer (final concentration: 300 nM), and 2.5 μL of DNA previously standardized at 1 ng/μL. PCR conditions were 95 °C for 2 min, and 40 cycles at 95 °C for 5 and 10 s at the optimized annealing temperature. A melt curve analysis was performed for SYBR-based qPCR assays under the following conditions: 1 min at 95 °C, 1 min at 55 °C, and 80 cycles of 0.5 °C increments (10 s each). A CFX96 Touch System (Bio-Rad Laboratories, Hercules, CA, USA) was used for all qPCR assays. Data are expressed as average values and standard deviations.

### 2.4. Computation and Statistical Analysis

Annotated Operational Taxonomic Units (OTUs) were imputed on a spreadsheet, together with diets, to allow and facilitate further statistical analysis. The annotates sequences from each sample and each taxonomic level were normalized to ‰ abundance profiles, already known as Relative Abundance (RA). Taxa with RA lower than 1‰ in more than half of the samples were excluded from the statistical analysis [23]. RAs were transformed into Absolute Abundances (AA), multiplying each datum with the quantification of total bacteria revealed by the qPCR for each sample. A non-parametric Kruskal–Wallis test was applied at the phylum, family and genera level of fecal and blood matrices, with Bonferroni correction for multiple comparison. A *p*-value below 0.05 was considered statistically significant, and below 0.1 was considered a trend. Principal Component Analysis (PCA) was used to analyze phylum, family and genera level of taxa within fecal and blood samples. Shannon and Evenness biodiversity indexes were calculated at the genus level of blood and gut microbiome [24]. Beta diversity was assessed with the Brian Curtis dissimilarity matrix and visualized by using principal coordinate analysis (PCoA) plot. Analysis of similarity (ANOSIM) was performed with the “Vegan” package in R (Version 3.2.1), to test whether the gut and the blood microbiome significantly differed between the three diets. All of these analyses were performed with XLSTAT [25].

## 3. Results

### 3.1. General Description of Blood Microbiome Related to Gut Microbiome

The collection of blood and fecal samples on the same day was conducted to investigate the possible relationship between blood microbiome and gut microbiome.

**Figure 1:**
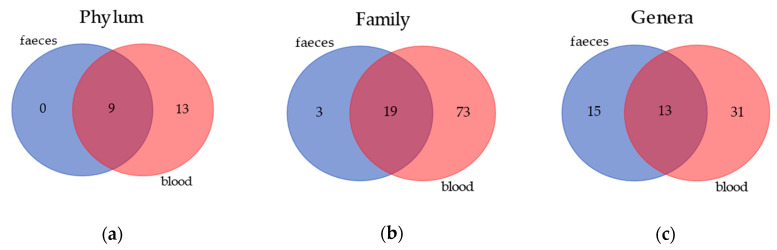
Similarity in bacterial community composition at phylum, family and genus levels in blood and feces of healthy dogs. (**a**) Venn diagrams showing the number of core phylum in blood and feces. Core phylum is defined as a phylum that is found in all dogs. (**b**) Venn diagrams showing the number of core families in blood and feces. Core family is defined as a phylum that is found in all dogs. (**c**) Venn diagrams showing the number of core genera in blood and feces. Core genera are defined as phyla that are found in all dogs.

Venn diagrams (Figure 1) show the results of the annotation comparison between blood and feces on three different taxa levels. As expected, the amount of bacteria in the blood was very low in comparison to feces; however, in the former matrix, the number of annotated taxa was much higher. Nevertheless, it is also notable that the blood and gut microbiomes share 9, 19 and 13 annotations at a phylum, family and genus level, respectively.

At the phylum level, gut and blood microbiome shared Actinobacteria, Bacteroidetes, Firmicutes, Fusobacteria and Proteobacteria (Table 1). Furthermore, the blood microbiome was characterized by four additional taxa, which were not shown in Table 1 because they were not relevant at the statistical analysis, due to the very low abundances detected for them. More interestingly, the blood microbiome is characterized by high quantities—in some cases even more than gut the microbiome—of Proteobacteria and Actinobacteria, in every diets considered in this study. Furthermore, Fusobacteria phylum on blood samples resulted in being significative different in BARF diet compared to the HOME diet, whilst dogs fed with a KIBBLE diet have intermediate values.

### 3.2. Characterization of Fecal Microbiome Related to Diets

At the family level, only the AA of Clostridiaceae, Coriobacteriaceae and Fusobacteriaceae resulted in significantly different copies between diets. In each of the thee families mentioned above, the higher abundance was detected in dogs fed with a BARF diet in comparison to dogs fed with a KIBBLE diet, whilst subjects that received a HOME diet had AA not significantly different from the other two diets. (Table 2). Lachnospiraceae family had very high AA in all dogs, and, although not significantly different between diets, these taxa showed a trend, with higher abundances in fecal samples of dogs fed with a KIBBLE diet, compared to subjects receiving a BARF or a HOME diet.

At the genus level (Table 3), again, *Clostridium* was significantly different in dogs fed with different diets. In particular, this genus had a higher AA in dogs fed with a BARF diet, compared to subjects receiving a HOME and a KIBBLE diet. The same trend was observed in the AA of *Collinsella*, which was significative higher in fecal samples of dogs fed the BARF diet. Although statistical analysis of the gut microbiome highlighted only these two genera, the other two genera showed a trend of AA between diets. The genus *Catenibacterium*, that is part of the Erysipelotrichaceae family (phylum Firmicutes), had higher AA in dogs fed with HOME diet, whilst it was decreased in fecal samples of dogs fed with KIBBLE diet, and those fed a BARF diet had the lowest abundances. Besides this taxa, also *Slackia* genus, part of the Coriobacteriaceae family (phylum Actinobacteria), showed a trend, having the highest AA in dogs fed with a BARF diet, whilst dogs of the KIBBLE-diet group had the lowest values.

**Figure 2:**
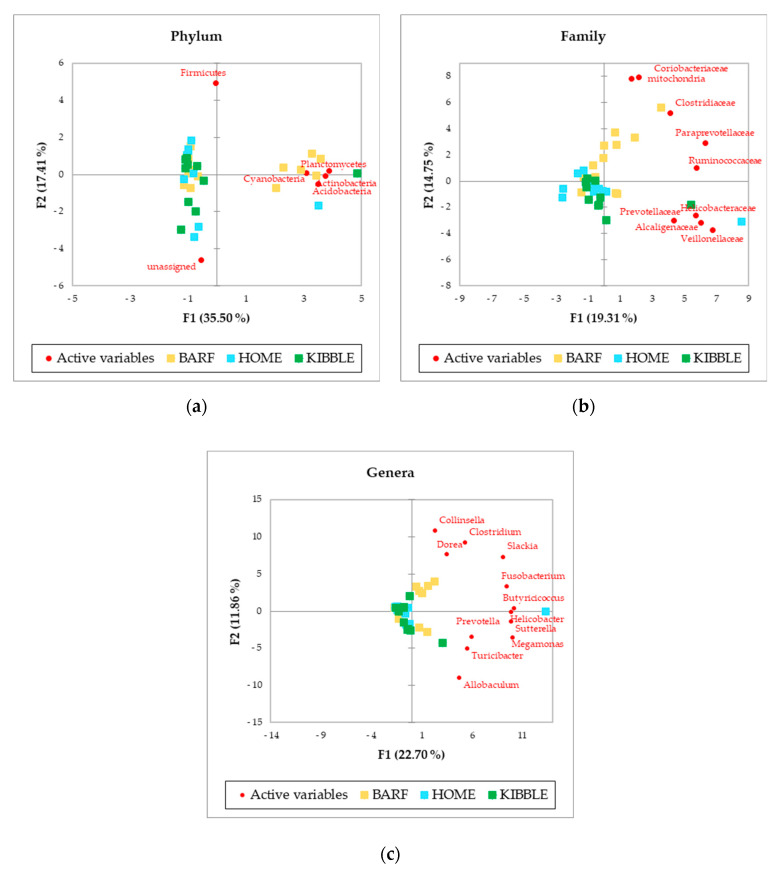
Principal Component Analysis (PCA) of bacteria Absolute Abundancies (AA) regarding (**a**) phylum level, (**b**) family level and (**c**) genus level on fecal samples of dogs fed with a raw-meat-based diet (BARF), a homemade based diet (HOME) and a commercial complete extruded diet (KIBBLE).

The PCA, performed with AA of phylum, family and genus level of the gut microbiome (Figure 2), showed a picture of the clusterization between the three groups of dogs based on their diets. In particular, the clearer clusterization was obtained for dogs fed with the BARF diet, at all the levels analyzed. Of greatest interest is the observation of some subjects that did not fit into the clusterization of their diet group.

### 3.3. Characterization of Blood Microbiome Related to Diets

A significant difference on blood microbiome in relation to different administered diet was observed. At the family level, the AA of Corynebacteriaceae, Fusobacteriaceae, Phyllobacteriaceae, Ruminococcaceae and Sphingomonadaceae resulted in being significantly different between the three diets. In particular, Fusobacteriaceae, Ruminococcaceae and Sphingomonadaceae showed the highest abundances in dogs fed with a BARF diet, compared to dogs fed with a KIBBLE and HOME diets (Table 4). The other taxa reported highlighted a trend with a *p*-value below 0.1. Moreover, in blood microbiome, Lachnospiraceae family was detected, and, as for gut microbiome, it had a higher AA in dogs fed with KIBBLE; on the contrary, intermediate values were shown in subjects receiving a BARF diet, whilst dogs of the HOME group diet had the lowest abundances.

At the genus level (Table 5), only *Corynebacterium*, belonging to the Corynebacteriaceae family (phylum Actinobacteria), resulted in significantly different amounts in dogs fed with different diets. In particular, this genus had higher AA in dogs fed with a BARF diet in comparison to subjects receiving a HOME diet and a KIBBLE diet. The other taxa reported in Table 5 highlighted a trend with a *p*-value below 0.1. Of interest were *Propionibacterium*, *Sphingomonas* and *Turicibacter* genera, which had higher AA compared to the other bacteria. *Propionibacterium*, part of the Propionibacteriaceae family (phylum Actinobacteria), was consistently higher in blood microbiome of subjects receiving a HOME diet, while it was detected in lower amounts in dogs fed with a BARF diet. In contrast, *Sphingomonas*, a genus of the Sphingomonadaceae family (phylum Proteobacteria), appeared to be higher in this latter group of dogs, compared to the HOME diet group. In the end, *Turicibacter*, belonging to the family of the Turicibacteriaceae (phylum Firmicutes), resulted in being higher in dogs fed with a KIBBLE diet, whilst the BARF group showed the lowest values.

**Figure 3:**
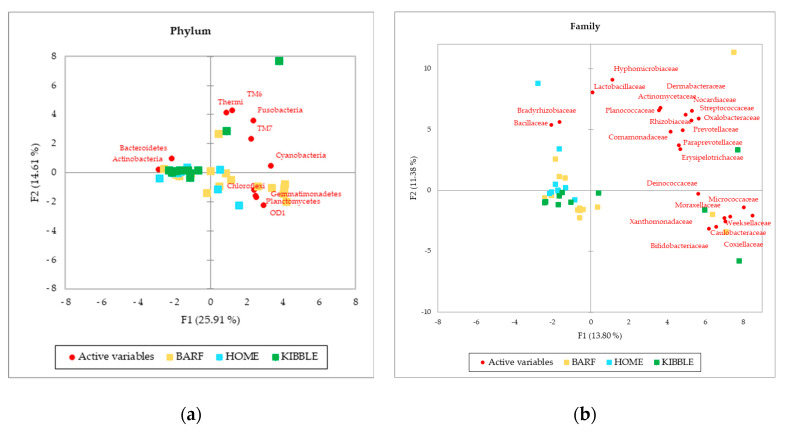

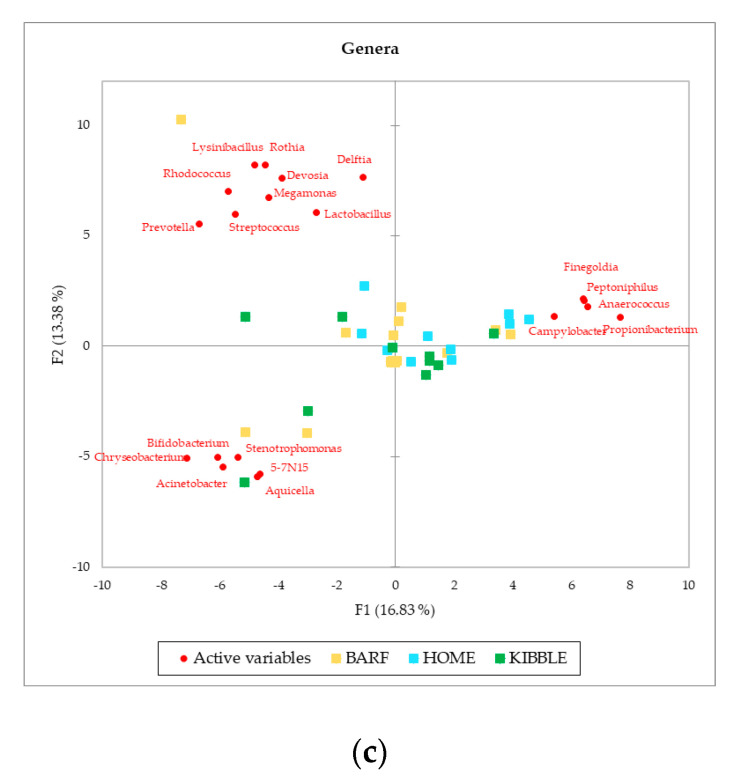
Principal Component Analysis (PCA) of bacteria Absolute Abundancies (AA) regarding (**a**) phylum level, (**b**) family level and (**c**) genera level on blood samples of dogs fed with a raw-meat-based diet (BARF), a homemade-based diet (HOME) and a commercial complete extruded diet (KIBBLE).

Although gut and blood microbiome showed differences in terms of taxa that can discriminate between dogs fed with different diets, a multivariate approach highlights that both types of microbiomes almost equally cluster the subjects on the base of what they eat. The PCA (Figure 3), performed with AA on phylum, family and genus level of the blood microbiome detected a clusterization of the three groups of dogs, based on their diets. The clearest clusterization was obtained with data from dogs fed with the BARF diet, and this result is better shown at the phylum level. Furthermore, in the blood microbiome, it was also observed that some dogs do not fit in the clusterization of their diet group.

### 3.4. Alpha and Beta Diversity of Gut and Blood Microbiome Related to Diets

Alpha and beta diversity levels were analyzed at the genus level of both gut and blood microbiome. Alpha diversity was calculated through the Shannon and Evenness indexes, and no significative differences were found in dogs fed with the three diets, in the microbial population of fecal and blood samples. Results are shown in Table 6.

**Figure 4:**
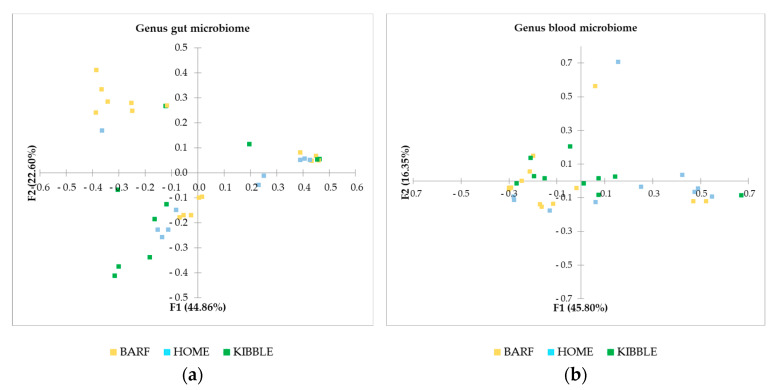
Beta diversity based on Bray–Curtis dissimilarity matrix at genus level: (**a**) gut microbiome and (**b**) blood microbiome. BARF, raw-meat diet; HOME, homemade diet; KIBBLE, complete extruded diet.

Beta diversity was computed through the Bray–Curtis dissimilarities distance matrix, at the genus level of both gut and blood microbiome. The PCoA (Figure 4) highlighted clear differences on gut microbiome of subjects fed with the three different diets. In particular, the dogs in the BARF group are very separated from the rest of the population, meaning that their microbial population is different from dogs fed a different type of diet. Although the separation of dogs is not distinct for gut microbiome case, in the blood microbiome, the clustering is appreciable.

## 4. Discussion

Several studies have pointed out that the gut microbiome is highly variable among healthy dogs [23] and can represent an individual fingerprint [8]. Several factors, other than different methodological approaches, affect the abundances of organisms in the gut microbiome, such as diet, sex, age and disease [26,27]. In humans, geographical variations, ethnicity, host genetic, immunity, lifestyle and dietary habits have been reported to affect gut microbiota [28]. In healthy dogs, variations of microbiome with age, from weaning to adulthood, were reported [27,29], with a stabilization of the core gut microbiota at maturity. A strong similarity between human and dog microbiota has been reported, suggesting that the same factors affecting gut microbial community in the former can act also in the latter [30].

Figure 2 showed a picture of the clustering between the three groups of dogs based on their diets. The more distinct clustering was appreciable for dogs fed with BARF diet, at phyla, family and genus level. Recently, a study on healthy dogs confirmed the role of diet on the gut microbiome [7], but also several other studies indicated the influence of diet on intestinal microbiome [2,4,23,31]. Beyond this result, it is interesting to observe that some subjects did not fit in the cluster of their dietary group. This evidence could be due to an unpredictable and discontinuous administration of foods not foreseen in the daily diet of the dogs, since they were not recruited from a kennel or a shelter, but from local veterinary clinics. The owners could have fed the dogs with rewards or fresh foods but not necessarily every day. For instance, the subjects receiving a KIBBLE diet, marked with green squares, were fed also with some homemade or some raw-meat-based diet, and this could also be possible for some dogs fed with a BARF or a HOME diet, where kibble could have been fed.

Moreover, the gut microbiome ecosystem has strong interactions with the environment and the genetics of the host. Vazquez-Baeza et al. (2016) [32] reported that the diversity and structure of microbial community, more than the variation of single taxon, could be used as a signature of the fecal microbiota to separate dogs with IBD from healthy dogs. The better characterization of the gut microbiome has been obtained, in the past, thanks to studies with subjects that lived in the same controlled environment, such as a shelter, kennel or university facilities, where the dogs received the same diet under strict control, either for a short or long period of time [5,29,31]. In this study, although the potential bias due to the effect of the different environments and of the owners can be claimed, it was still possible to separate dogs based on their diet. Moreover, the characterization of gut microbiome with a multivariate approach allowed the identification of those subjects for which diet was mixed in terms of administered type.

Another aim of the study was to investigate if gut microbes can translocate to the blood crossing the gut wall. The so called “leaky gut” condition is widely studied in humans and dogs and often is related to inflammatory bowel diseases and related enteropathies [33,34,35,36], but this functional deficiency should not happen in healthy animals. In the present study, we analyzed the presence of bacterial DNA in blood of healthy animals, and, surprisingly, several taxa were detected. Blood has been traditionally considered to be a sterile environment, but some evidences for bacterial presence in various domesticated mammals and birds [11,12] and in humans [13,14,15] do exist. Despite the difficulties to cultivate blood bacteria, several studies reported the successful growth of numerous bacteria from blood of healthy individuals confirmed by microscopic observation [18,37]. Even if the majority of taxa annotated in blood was present at a very low abundances, they were detectable. One of the hypotheses is that the bacteria populating the blood are in a “dormient phase” [38], and this would explain why there is a microbial population even in healthy subjects and also why this condition is not pathogenic. The hypothesis is that, occasionally, some of these bacteria “wake up” and reproduce, becoming active again and being transported to various tissues and organs of the body, thus inducing a state of chronic disease [39]. To the best of our knowledge, this is the first study characterizing canine blood microbiome. External factors, such as contamination of reagents and blood with external bacteria during the sampling, could have led to an artifactual appearance of microbiome into the blood. For what the reagents and the sequencing pipeline is concerned, a mock bacterial community was used as internal standard to validate the methodology, and the results confirmed the lack of contamination.

Interestingly, the number of bacterial taxa annotated for blood samples was much higher compared to fecal samples, especially at a family level, with 92 taxa annotated in blood and 22 in feces (Figure 1). The blood bacterial taxa found in this study are in line with the literature found for humans [16]. The most detected bacteria in blood (Table 1) were Proteobacteria and Actinobacteria, which differed consistently from the predominant phyla of the gut microbiome, that were Firmicutes and Bacteroidetes [2,40]. This difference between blood and feces could be explained by the role of filter played by intestinal and immune cells, which could have limited the translocation of certain bacteria. The recognition of “good” or “bad” microorganisms is due to the presence of dendritic cells in the gut [41], although environmental factors can shift commensal bacteria to pathogenic bacteria, causing a disruption at a variable extent of tight junction and a leaky gut. Furthermore, tissues and organs, such as skin, oral cavity and nasal or vaginal mucosa, can probably contribute to the bacterial DNA present in the blood [42]. Although the microbial composition in the feces was not equal to those in the blood, in terms of abundances and presence of certain taxa, the clustering of dogs in the three diets was comparable. The PCA multivariate analysis allowed to separate the dogs by using the gut microbiome (Figure 2), and almost the same results were obtained by using the blood microbial community (Figure 3).

## 5. Conclusions

This study confirmed that diet is a factor driving the shift of gut microbial population in dogs, and researches in this direction are still needed to better clarify the association between this factor and gut microbiome.

A limited number of research studies have been published on blood microbiota in humans, and this is the first evidence of the presence of a bacterial population in the blood of healthy dogs. From our results, we can speculate that blood microbiome, or at least a part of it, can derive from the translocation of some gut bacteria, and it consequently could be associated with a shift of the intestinal microbial population. These preliminary results deserve further studies, including also dogs suffering of gastrointestinal diseases; however, if confirmed, the results pave the way for the use of blood microbiome for diagnostic purposes.

## Figures and Tables

**Table 1:** Mean and standard deviation of phylum Absolute Abundances (AA), expressed in 16S copies DNA/g bacteria, characterizing feces and blood of subjects fed with three different diets. ^a,b^ on the same row denotes differences between means for *p*-value < 0.05.

		BARF			HOME			KIBBLE		
		Mean		SD	Mean		SD	Mean		SD
Actinobacteria	feces	65.0		83.9	18.2		30.8	21.6		32.2
	blood	8.8		10.2	21.2		17.2	12.7		7.2
Bacteroidetes	feces	97.3		119.8	69.7		97.2	106.1		89.1
	blood	2.8		5.1	3.1		3.9	10.9		8.2
Firmicutes	feces	1062.3		368.0	1064.2		474.6	896.3		345.7
	blood	24.6		11.9	30.7		25.9	31.0		12.8
Fusobacteria	feces	100.8		99.9	93.7		126.2	75.9		141.0
	blood	0.2	^b^	0.2	0.0	^a^	0.0	0.1	^ab^	0.4
Proteobacteria	feces	10.4		10.9	60.1		83.7	57.4		128.8
	blood	18.2		14.5	13.3		21.5	17.6		14.8
OD1	feces	n.a.			n.a.			n.a.		
	blood	1.9		2.6	0.8		2.6	0.0		0.1

BARF, raw-meat-based diet; HOME, homemade diet; KIBBLE, complete extruded diet.

**Table 2:** Kruskal–Wallis non-parametric test results of the family Absolute Abundances (AA), expressed in 16S copies DNA/g bacteria, characterizing fecal samples of subjects fed with three different diets. Mean and standard deviation (SD) are reported; ^a,b^ on the same row denotes differences between means for *p*-value < 0.05.

	BARF			HOME			KIBBLE			
	Mean		SD	Mean		SD	Mean		SD	*p*-Value
Clostridiaceae	446.6	^b^	364.5	229.5	^ab^	268.3	175.4	^a^	195.7	0.045
Coriobacteriaceae	75.5	^b^	75.7	29.1	^ab^	37.1	19.6	^a^	35.7	0.021
Fusobacteriaceae	132.5	^b^	165.1	75.0	^ab^	79.8	16.0	^a^	20.2	0.015
Lachnospiraceae	304.8		173.3	338.2		291.5	428.3		247.9	0.469

BARF, raw-meat diet; HOME, homemade diet; KIBBLE, complete extruded diet.

**Table 3:** Kruskal–Wallis non-parametric test results of the genus Absolute Abundances (AA), expressed in 16S copies DNA/g bacteria, characterizing fecal samples of subjects fed with three different diets’ mean and standard deviation (SD) are reported; ^a,b^ on the same row denotes differences between means for *p*-value < 0.05.

	BARF			HOME			KIBBLE			
	Mean		SD	Mean		SD	Mean		SD	*p*-Value
*Catenibacterium*	2.1		8.3	18.5		31.7	6.9		15.1	0.069
*Clostridium*	389.0	^b^	389.4	139.9	^a^	227.8	122.7	^ab^	119.7	0.030
*Collinsella*	56.4	^b^	74.6	11.8	^ab^	10.8	2.5	^a^	4.9	0.022
*Slackia*	4.7		7.0	3.3		10.4	0.1		0.1	0.071

BARF, raw-meat diet; HOME, homemade diet; KIBBLE, complete extruded diet.

**Table 4:** Kruskal–Wallis non-parametric test results of the family Absolute Abundances (AA), expressed in 16S copies DNA/g bacteria, characterizing blood samples of subjects fed with three different diets mean and standard deviation (SD) are reported; ^a,b^ on the same row denotes differences between means for *p*-value < 0.05.

	BARF			HOME			KIBBLE			
	Mean		SD	Mean		SD	Mean		SD	*p*-Value
Bifidobacteriaceae	0.2		0.2	0.0		0.0	0.3		0.7	0.066
Coriobacteriaceae	0.7		2.0	0.0		0.0	0.6		1.7	0.070
Corynebacteriaceae	0.4	^ab^	0.6	0.7	^b^	0.6	0.0	^a^	0.0	0.003
Fusobacteriaceae	0.2	^b^	0.2	0.0	^a^	0.0	0.1	^ab^	0.4	0.027
Lachnospiraceae	3.5		3.6	0.3		0.5	3.8		6.5	0.054
Phyllobacteriaceae	0.0	^a^	0.0	0.4	^b^	0.8	0.0	^ab^	0.0	0.016
Propionibacteriaceae	6.0		10.0	19.5		16.0	9.9		9.7	0.057
Ruminococcaceae	4.9	^b^	5.8	0.1	^a^	0.2	1.6	^ab^	2.8	0.031
Sphingomonadaceae	10.2	^b^	11.3	0.6	^a^	1.6	1.9	^ab^	2.9	0.045
Turicibacteriaceae	0.5		1.5	1.4		1.9	2.8		4.0	0.054

BARF, raw-meat diet; HOME, homemade diet; KIBBLE, complete extruded diet.

**Table 5:** Kruskal–Wallis non-parametric test results of the genus Absolute Abundances (AA), expressed in 16S copies/g, characterizing blood samples of subjects fed with three different diets. Mean and standard deviation (SD) are reported; ^a,b^ on the same row denotes differences between means for *p*-value < 0.05. BARF, raw-meat diet; HOME, homemade diet; KIBBLE, complete extruded diet.

	BARF			HOME			KIBBLE			
	Mean		SD	Mean		SD	Mean		SD	*p*-Value
Corynebacterium	0.4	^ab^	0.6	0.7	^b^	0.6	0.0	^a^	0.0	0.003
Delftia	0.5		1.6	1.1		1.3	0.8		0.8	0.058
Propionibacterium	6.0		10.0	19.5		16.0	9.9		9.7	0.057
Sedimentibacter	0.0		0.0	0.3		0.9	0.7		1.2	0.069
Sphingomonas	9.7		11.5	0.3		0.8	1.5		1.9	0.071
Turicibacter	0.5		1.5	1.4		1.9	2.8		4.0	0.054

**Table 6:** Shannon (H’) and Evenness (J) indexes of gut and blood microbiome at genus level, characterizing dogs fed three different diets. Mean and standard deviation (SD) are reported.

		BARF		HOME		KIBBLE	
		Mean	SD	Mean	SD	Mean	SD
H’	feces	1.179	0.560	1.115	0.611	1.312	0.733
	blood	1.295	0.407	1.174	0.726	1.612	0.210
J	feces	0.358	0.170	0.338	0.185	0.398	0.223
	blood	0.342	0.108	0.310	0.192	0.426	0.055

BARF, raw-meat diet; HOME, homemade diet; KIBBLE, complete extruded diet.

## References

[1] Sandri M., Manfrin C., Pallavicini A., Stefanon B. (2014). Microbial biodiversity of the liquid fraction of rumen content from lactating cows. Animal.

[2] Deng P., Swanson K.S. (2015). Gut microbiota of humans, dogs and cats: Current knowledge and future opportunities and challenges. Br. J. Nutr..

[3] Pilla R., Suchodolski J.S. (2020). The Role of the Canine Gut Microbiome and Metabolome in Health and Gastrointestinal Disease. Front. Vet. Sci..

[4] Jha A.R., Shmalberg J., Tanprasertsuk J., Perry L., Massey D., Honaker R.W. (2020). Characterization of gut microbiomes of household pets in the United States using a direct-to-consumer approach. PLoS ONE.

[5] Forster G.M., Stockman J., Noyes N., Heuberger A.L., Broeckling C.D., Bantle C.M., Ryan E.P. (2018). A Comparative Study of Serum Biochemistry, Metabolome and Microbiome Parameters of Clinically Healthy, Normal Weight, Overweight, and Obese Companion Dogs. Top. Companion Anim. Med..

[6] Hand D., Wallis C., Colyer A., Penn C.W. (2013). Pyrosequencing the Canine Faecal Microbiota: Breadth and Depth of Biodiversity. PLoS ONE.

[7] Scarsella E., Stefanon B., Cintio M., Licastro D., Sgorlon S., Monego S.D., Sandri M. (2020). Learning machine approach reveals microbial signatures of diet and sex in dog. PLoS ONE.

[8] Garcia-Mazcorro J.F., Barcenas-Walls J.R., Suchodolski J.S., Steiner J.M. (2017). Molecular assessment of the fecal microbiota in healthy cats and dogs before and during supplementation with fructo-oligosaccharides (FOS) and inulin using high-throughput 454-pyrosequencing. PeerJ.

[9] Arumugam M., Raes J., Pelletier E., Le Paslier D., Yamada T., Mende D.R., Fernandes G.R., Tap J., Bruls T., Batto J.-M. (2011). Enterotypes of the human gut microbiome. Nature.

[10] Petersen C., Round J.L. (2014). Defining dysbiosis and its influence on host immunity and disease. Cell. Microbiol..

[11] Mandal R.K., Jiang T., Al-Rubaye A.A., Rhoads D.D., Wideman R.F., Zhao J., Pevzner I., Kwon Y.M. (2016). An investigation into blood microbiota and its potential association with Bacterial Chondronecrosis with Osteomyelitis (BCO) in Broilers. Sci. Rep..

[12] Vientós-Plotts A.I., Ericsson A.C., Rindt H., Grobman M.E., Graham A., Bishop K., Cohn L.A., Reinero C.R. (2017). Dynamic changes of the respiratory microbiota and its relationship to fecal and blood microbiota in healthy young cats. PLoS ONE.

[13] Li Q., Wang C., Tang C., Zhao X., He Q., Li J. (2018). Identification and Characterization of Blood and Neutrophil-Associated Microbiomes in Patients with Severe Acute Pancreatitis Using Next-Generation Sequencing. Front. Cell. Infect. Microbiol..

[14] Whittle E., Leonard M.O., Harrison R., Gant T.W., Tonge D.P. (2019). Multi-Method Characterization of the Human Circulating Microbiome. Front. Microbiol..

[15] Qiu J., Zhou H., Jing Y., Dong C. (2019). Association between blood microbiome and type 2 diabetes mellitus: A nested case-control study. J. Clin. Lab. Anal..

[16] Païssé S., Valle C., Servant F., Courtney M., Burcelin R., Amar J., Lelouvier B. (2016). Comprehensive description of blood microbiome from healthy donors assessed by 16S targeted metagenomic sequencing. Transfusion.

[17] Cogen A.L., Nizet V., Gallo R.L. (2008). Skin microbiota: A source of disease or defence?. Br. J. Dermatol..

[18] Potgieter M., Bester J., Kell D.B., Pretorius E. (2015). The dormant blood microbiome in chronic, inflammatory diseases. FEMS Microbiol. Rev..

[19] Mercier R., Kawai Y., Errington J. (2014). General principles for the formation and proliferation of a wall-free (L-form) state in bacteria. eLife.

[20] Klindworth A., Pruesse E., Schweer T., Peplies J., Quast C., Horn M., Glöckner F.O. (2013). Evaluation of general 16S ribosomal RNA gene PCR primers for classical and next-generation sequencing-based diversity studies. Nucleic Acids Res..

[21] Bolyen E., Rideout J.R., Dillon M.R., Bokulich N.A., Abnet C.C., Al-Ghalith G.A., Alexander H., Alm E.J., Arumugam M., Asnicar F. (2019). Reproducible, interactive, scalable and extensible microbiome data science using QIIME 2. Nat. Biotechnol..

[22] AlShawaqfeh M.K., Wajid B., Minamoto Y., Markel M., Lidbury J.A., Steiner J.M., Serpedin E., Suchodolski J.S. (2017). A dysbiosis index to assess microbial changes in fecal samples of dogs with chronic inflammatory enteropathy. FEMS Microbiol. Ecol..

[23] Sandri M., Sgorlon S., Conte G., Serra A., Monego S.D., Stefanon B. (2019). Substitution of a commercial diet with raw meat complemented with vegetable foods containing chickpeas or peas affects faecal microbiome in healthy dogs. Ital. J. Anim. Sci..

[24] Sandri M., Sgorlon S., Scarsella E., Stefanon B. (2020). Effect of different starch sources in a raw meat-based diet on fecal microbiome in dogs housed in a shelter. Anim. Nutr..

[25] Addinsoft (2020). XLSTAT Statistical and Data Analysis Solution.

[26] Cintio M., Scarsella E., Sgorlon S., Sandri M., Stefanon B. (2020). Gut Microbiome of Healthy and Arthritic Dogs. Vet. Sci..

[27] Vilson Å., Ramadan Z., Li Q., Hedhammar Å., Reynolds A., Spears J., Labuda J., Pelker R., Björkstén B., Dicksved J. (2018). Disentangling factors that shape the gut microbiota in German Shepherd dogs. PLoS ONE.

[28] Gupta V.K., Paul S., Dutta C. (2017). Geography, Ethnicity or Subsistence-Specific Variations in Human Microbiome Composition and Diversity. Front. Microbiol..

[29] Ribeiro É.D.M., Peixoto M.C., Putarov T.C., Monti M., Pacheco P.D.G., Loureiro B.A., Pereira G.T., Carciofi A.C. (2019). The effects of age and dietary resistant starch on digestibility, fermentation end products in faeces and postprandial glucose and insulin responses of dogs. Arch. Anim. Nutr..

[30] Coelho L.P., Kultima J.R., Costea P.I., Fournier C., Pan Y., Czarnecki-Maulden G., Hayward M.R., Forslund S.K., Schmidt T.S.B., Descombes P. (2018). Similarity of the dog and human gut microbiomes in gene content and response to diet. Microbiome.

[31] Beloshapka A.N., Dowd S.E., Suchodolski J.S., Steiner J.M., Duclos L., Swanson K.S. (2013). Fecal microbial communities of healthy adult dogs fed raw meat-based diets with or without inulin or yeast cell wall extracts as assessed by 454 pyrosequencing. FEMS Microbiol. Ecol..

[32] Vázquez-Baeza Y., Hyde E.R., Suchodolski J.S., Knight R. (2016). Dog and human inflammatory bowel disease rely on overlapping yet distinct dysbiosis networks. Nat. Microbiol..

[33] Ridyard A.E., Brown J.K., Rhind S.M., Else R.W., Simpson J.W., Miller H.R.P. (2007). Apical Junction Complex Protein Expression in the Canine Colon: Differential Expression of Claudin-2 in the Colonic Mucosa in Dogs with Idiopathic Colitis. J. Histochem. Cytochem..

[34] Suchodolski J.S. (2016). Diagnosis and interpretation of intestinal dysbiosis in dogs and cats. Vet. J..

[35] Stewart A.S., Pratt-Phillips S., Gonzalez L.M. (2017). Alterations in Intestinal Permeability: The Role of the “Leaky Gut” in Health and Disease. J. Equine Vet. Sci..

[36] Tizard I.R., Jones S.W. (2018). The Microbiota Regulates Immunity and Immunologic Diseases in Dogs and Cats. Vet. Clin. Small Anim. Pract..

[37] Damgaard C., Magnussen K., Enevold C., Nilsson M., Tolker-Nielsen T., Holmstrup P., Nielsen C.H. (2015). Viable Bacteria Associated with Red Blood Cells and Plasma in Freshly Drawn Blood Donations. PLoS ONE.

[38] Kell D.B., Kaprelyants A.S., Weichart D.H., Harwood C.R., Barer M.R. (1998). Viability and activity in readily culturable bacteria: A review and discussion of the practical issues. Antonie Van Leeuwenhoek.

[39] Kell D.B., Pretorius E. (2015). On the translocation of bacteria and their lipopolysaccharides between blood and peripheral locations in chronic, inflammatory diseases: The central roles of LPS and LPS-induced cell death. Int. Biol..

[40] Turnbaugh P.J., Ley R.E., Mahowald M.A., Magrini V., Mardis E.R., Gordon J.I. (2006). An obesity-associated gut microbiome with increased capacity for energy harvest. Nature.

[41] Rimoldi M., Chieppa M., Salucci V., Avogadri F., Sonzogni A., Sampietro G.M., Nespoli A., Viale G., Allavena P., Rescigno M. (2005). Intestinal immune homeostasis is regulated by the crosstalk between epithelial cells and dendritic cells. Nat. Immunol..

[42] Costello E.K., Lauber C.L., Hamady M., Fierer N., Gordon J.I., Knight R. (2009). Bacterial Community Variation in Human Body Habitats Across Space and Time. Science.

